# Mitochondrial DNA in inflammation and immunity

**DOI:** 10.15252/embr.201949799

**Published:** 2020-03-23

**Authors:** Joel S Riley, Stephen WG Tait

**Affiliations:** ^1^ Cancer Research UK Beatson Institute Glasgow UK; ^2^ Institute of Cancer Sciences University of Glasgow Glasgow UK

**Keywords:** cell death, inflammation, immunity, mitochondria, mtDNA, Autophagy & Cell Death, Immunology

## Abstract

Mitochondria are cellular organelles that orchestrate a vast range of biological processes, from energy production and metabolism to cell death and inflammation. Despite this seemingly symbiotic relationship, mitochondria harbour within them a potent agonist of innate immunity: their own genome. Release of mitochondrial DNA into the cytoplasm and out into the extracellular milieu activates a plethora of different pattern recognition receptors and innate immune responses, including cGAS‐STING, TLR9 and inflammasome formation leading to, among others, robust type I interferon responses. In this Review, we discuss how mtDNA can be released from the mitochondria, the various inflammatory pathways triggered by mtDNA release and its myriad biological consequences for health and disease.

## Graphical abstract

Mitochondrial DNA is increasingly recognized as a potent agonist of innate immunity. This review discusses how mitochondrial DNA can be released from mitochondria and trigger the activation of different pro‐inflammatory signaling pathways.

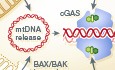

**Glossary**
5hmC: 5‐Hydroxymethylcytosine
5mC: 5‐Methylcytosine
AGS: Aicardi–Goutieres syndrome
AIM2: Absent in melanoma 2
APC: Antigen‐presenting cell
ASC: Apoptosis‐associated speck‐like protein containing a CARD
ATP: Adenosine triphosphate
BAK: Bcl‐2 homologous antagonist/killer
BAX: Bcl‐2‐associated X protein
BID: BH3 interacting‐domain death agonist
CARD: Caspase activation and recruitment domain
CD47: Cluster of differentiation 47
CDN: Cyclic dinucleotide
cGAMP: Cyclic guanosine monophosphate–adenosine monophosphate
cGAS: Cyclic GMP‐AMP synthase
CLR: C‐type lectin receptor
CMPK2: Cytidine/Uridine monophosphate kinase 2
DAMP: Damage‐associated molecular pattern
DC: Dendritic cell
DNase: Deoxyribonuclease
dsDNA: Double‐stranded DNA
ER: Endoplasmic reticulum
EV: Extracellular vesicle
GTP: Guanosine‐5′‐triphosphate
HMGB1: High‐mobility group protein 1
HSV‐1: Herpes simplex virus‐1
IAP: Inhibitor of apoptosis protein
IFNAR: Interferon‐α/β receptor
IFN‐β: Interferon‐β
IFN‐γ: Interferon‐γ
IL‐18: Interleukin‐18
IL‐1R: Interleukin‐1 receptor
IL‐1β: Interleukin‐1β
IL‐6: Interleukin‐6
IRF3: Interferon regulatory factor 3
ISG: Interferon‐stimulated gene
K+: Potassium
LPS: Lipopolysaccharide
LRR: Leucine‐rich repeat
MAPK: Mitogen‐activated protein kinase
MAVS: Mitochondrial anti‐viral signalling protein
MDA5: Melanoma differentiation‐associated protein 5
MEF: Mouse embryonic fibroblast
MiDAS: Mitochondrial dysfunction‐associated senescence
MI: Myocardial infarction
MOMP: Mitochondrial outer membrane permeabilisation
mPTP: Mitochondrial permeability transition pore
mtDNA: Mitochondrial DNA
NASH: Non‐alcoholic fatty liver disease
NET: Neutrophil extracellular trap
NF‐κB: Nuclear factor kappa‐light‐chain‐enhancer of activated B cells
NLRC4: NLR Family CARD Domain Containing 4
NLR: Nucleotide oligomerisation domain‐like receptor
NLRP1: NLR Family Pyrin Domain Containing 1
NLRP3: NACHT, LRR and PYD domain‐containing protein 3
NOD: Nucleotide oligomerisation domain
ODN: Oligodeoxynucleotide
OPA1: Optic Atrophy 1 Mitochondrial Dynamin Like GTPase
PAMP: Pathogen‐associated molecular pattern
pDC: Plasmacytoid dendritic cell
PD‐L1: Programmed death‐ligand 1
PINK1: Phosphatase and tensin homolog‐induced kinase 1
PMA: Phorbol 12‐myristate 13‐acetate
PNPase: Polynucleotide phosphorylase
PRR: Pattern recognition receptor
PYD: Pyrin domain
RAGE: Receptor for advanced glycation endproducts
RIG‐I: Retinoic acid‐inducible gene I
RIP1: Receptor‐interacting serine/threonine‐protein kinase 1
RLR: Retinoic acid‐inducible gene‐I‐like receptors
RNP IC: Ribonucleotide immune complex
ROS: Reactive oxygen species
SAMDH1: Sterile alpha motif domain and HD domain‐containing protein 1
SIRS: Systemic inflammatory response syndrome
SLE: Systemic lupus erythematosus
ssDNA: Single‐stranded DNA
STING: Stimulator of interferon genes
SUV3: Suppressor of Var1
TBK1: TANK‐binding kinase 1
TFAM: Transcription factor A, mitochondrial
TLR9: Toll‐like receptor 9
TLR: Toll‐like receptor
TNF: Tumour necrosis factor
TREX1: Three Prime Repair Exonuclease 1
tRNA: Transfer RNA
VDAC: Voltage‐dependent anion channel

## Introduction

Serving as a first line of defence, the innate immune system guards us against a plethora of insults and invading microorganisms. Infection by pathogenic agents is detected in cells by pattern recognition receptors (PRRs) which recognise specific pathogen‐associated molecular patterns (PAMPs). PRRs can be broadly classified into four distinct groups: NOD‐like receptors (NLRs), Toll‐like receptors (TLRs), retinoic acid‐inducible gene‐I (RIG‐I)‐like receptors (RLRs) and C‐type lectin receptors (CLRs) 1. Upon detection of a PAMP, PRRs initiate a multitude of different signalling pathways, which culminate in the up‐regulation of various type I interferons, pro‐inflammatory chemokines and cytokines. These prime the adaptive immune system and create a hostile environment for the microorganism in which to survive. Additionally, damage‐associated molecular patterns (DAMPs) are immune triggers that arise from the cell itself, such as proteins or DNA, and can activate innate immune pathways 2.

Mitochondria first appeared in eukaryotic cells about two billion years ago as α‐proteobacterium, in what is thought to be an endosymbiotic relationship 3, 4. Over time, these bacteria evolved to become the much‐studied organelle that we know today, playing crucial roles in metabolism, calcium homeostasis and cell death. Nevertheless, they have maintained an independent genome, which encodes 37 genes, comprised of 13 mRNAs forming key components of the oxidative phosphorylation system, in addition to 2 ribosomal RNA components and 22 tRNAs 3, 4. An estimated 1,000 proteins are located in the mitochondria, all of which, except those encoded by mtDNA, are translated in the cytosol and imported into the mitochondria 5.

Mitochondrial DNA itself is a circular molecule of double‐stranded (ds)DNA. Transcription of both the heavy and light strand results in long, full‐length transcripts which are processed by RNase enzymes to produce mature mRNA, tRNA and ribosomal RNA. In mammals, the polymerase responsible for mtDNA replication is DNA polymerase γ, but as POLγ cannot replicate dsDNA, the DNA helicase Twinkle is required to act directly before to unwind the DNA structure. Newly synthesised single‐stranded (ss)DNA is bound by mitochondrial single‐stranded DNA‐binding protein to prevent secondary structure formation and attack by nucleases. Mitochondrial DNA replication has recently been reviewed extensively elsewhere 6; here, we focus on the unique aspects of mtDNA which make it immunostimulatory. We will then discuss how mtDNA which is ejected from the mitochondria under specific circumstances can activate different innate immune pathways, including cGAS‐STING signalling, inflammasomes and Toll‐like receptors. We will also focus on the role of mtDNA in the formation of neutrophil extracellular traps (NETs) and the transfer of mtDNA between cells.

## Mitochondrial DNA as a stimulator of the immune system

Potentially stemming from its bacterial origin, mitochondrial DNA is sensed as “foreign”, suggesting that it is seen differently to “self” DNA in cells. One example of this can be seen in its methylation status, where many studies have reported mtDNA to be hypomethylated compared to nuclear DNA 7, 8, despite the presence of DNA methyltransferases in the mitochondria 9, 10. Some groups have reported aberrant methylation patterns of mtDNA, including 5‐methylcytosine (5mC) and 5‐hydroxymethylcytosine (5hmC) at CpG motifs 9, 10, 11, 12, 13, 14, although others have proposed technical limitations to this work and using more sensitive techniques report that mtDNA is devoid of CpG methylation 15. Clearly, more effort is required in determining the precise degree of methylation in mtDNA, but if studies showing an absence of CpG methylation are correct, then mtDNA would harbour unmethylated CpG motifs similar to bacterial DNA, which could potentially activate pattern recognition receptors such as TLR9, absent in melanoma 2 (AIM2) and cGAS 15, 16, 17, 18. Mitochondrial DNA replication and transcription itself may represent a rich source of potential activators of DNA pattern recognition receptors; for example, RNA:DNA hybrids form during transcription, in addition to long stretches of ssDNA and R‐loops composed of RNA:DNA hybrids with a non‐template ssDNA which can be recognised by cGAS 16.

Mitochondrial DNA exists in the mitochondrial matrix in close proximity to the electron transport chain, a major source of reactive oxygen species. Due to this, it is particularly vulnerable to oxidation, resulting in mtDNA mutations which can contribute to the pathogenesis of cancer 17, diabetes 18 and ageing 19. It was thought the cell had limited capacity to repair mtDNA; however, multiple repair pathways are now well characterised 20. Mitochondrial DNA is often schematically represented as a plasmid structure; however, this is an over‐simplification. Rather, super‐resolution imaging has revealed that it is densely compacted into nucleoids consisting of one copy of mtDNA and a number of different proteins 21, the most notable of which is mitochondrial transcription factor A (mtTFA, commonly referred to as TFAM). It might be assumed that the compaction of mtDNA into protein structures shields DNA from recognition, but this is not the case as we shall discuss further in this Review, and in fact, a number of studies have shown that TFAM itself might be immunostimulatory 13, 14.

**Figure 1:**
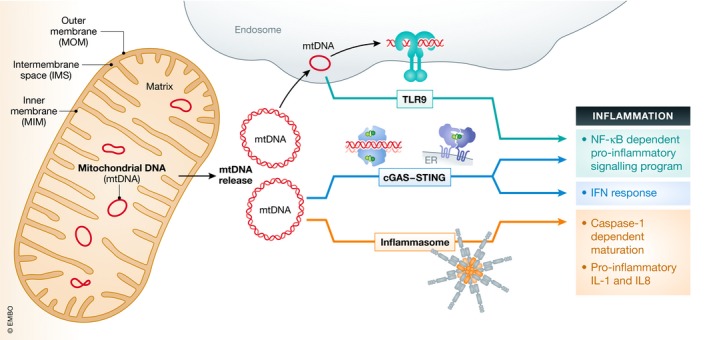
Overview of pro‐inflammatory signalling pathways engaged by mitochondrial DNA Mitochondrial DNA (mtDNA) can trigger various pro‐inflammatory signalling pathways by endosomal localised TLR9 or via cytosolic cGAS‐STING or via cytosolic inflammasome (AIM2 or NLRP3). Top: TLR9 binds mtDNA in the endosome eliciting an NF‐κB‐dependent pro‐inflammatory signalling program. Middle: cGAS recognises mtDNA in the cytosol and activates endoplasmic reticulum (ER)‐localised STING triggering an interferon response. Bottom: mtDNA‐dependent inflammasome activity leads to caspase‐1‐dependent maturation or pro‐inflammatory IL‐1 and IL‐8.

In a landmark study in 2004, Collins *et al* 22 found that injecting mtDNA into the joints of mice resulted in localised inflammation and arthritis. Further investigation revealed that the inflammation was dependent on the presence of oxidatively damaged bases in the mtDNA, as injection of an oligodeoxynucleotide (ODN) with the same sequence but without the oxidised residue had no effect. The observation that mtDNA can elicit potent immune responses opened a whole new field of research, and it is now appreciated that mtDNA can stimulate many PRRs, including cGAS, TLR9 and inflammasomes (Fig 1). Release of mtDNA from mitochondria and subsequent recognition by PRRs occurs during many cellular processes, including infection, cell death and neurodegeneration, and this will be the focus of the rest of this Review.

## mtDNA‐dependent activation of cGAS‐STING signalling

### mtDNA release in infection

**Figure 2:**
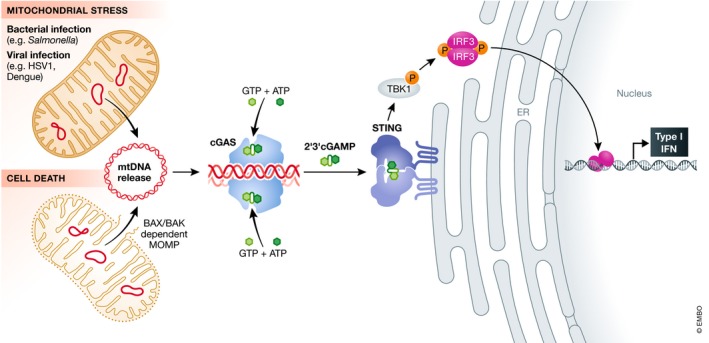
mtDNA‐dependent activation of cGAS‐STING signalling Various mitochondrial stresses including bacterial or viral infection can lead to mtDNA release. Alternatively, activation of BAX and BAK leads to outer mitochondrial membrane permeabilisation (MOMP) and mtDNA release. Once cytoplasmic, mtDNA can bind the DNA sensing protein cGAS that catalyses the production of the secondary messenger 2′3′ cyclic GMP–AMP (2′3′cGAMP) from ATP and GTP. cGAMP binds the adaptor molecule STING on the ER leading to activation of TBK1 kinase. Active TBK1 phosphorylates the transcription factor IRF3 initiating a type I interferon response.

Through necessity, cells have evolved elegant systems to detect the presence of invading pathogenic DNA. Cyclic GMP‐AMP synthase (cGAS) is one such direct detector, which binds dsDNA to form a dimer 23, 24. cGAS then undergoes a conformational change which facilitates the conversion of ATP and GTP into 2′3′‐cyclic GMP‐AMP (cGAMP) 25, 26, 27, 28, 29, 30, 31. cGAMP is a second messenger, which binds the endoplasmic reticulum (ER)‐resident protein stimulator of interferon genes (STING) inducing a conformational change in its C‐terminal tail. TANK‐binding kinase 1 (TBK1) is recruited to STING which phosphorylates it and the transcription factor interferon regulatory factor 3 (IRF3), eliciting the transcription of hundreds of interferon stimulatory genes (ISGs) that are potently anti‐viral 32 (Fig 2). cGAS was assumed to be primarily cytosolic to avoid persistent activation by self‐DNA in the nucleus, but recent work has shown it to be present in the nucleus 33, 34 and at the plasma membrane 35. A recent attempt to resolve these discrepancies by Volkmann *et al* 36 reveals a more complex model than the cytosolic DNA sensing paradigm. The authors show that the majority of cGAS protein is nuclear, and they propose a model where cGAS must be “desequestered” prior to its full activation. However, it remains unclear how cytosolic DNA can be detected by cGAS, if cGAS is tethered in the nuclear compartment. Three independent studies were the first to show that mtDNA released from mitochondria is able to activate cGAS‐STING signalling 37, 38, 39. White *et al* and Rongvaux *et al* explored mtDNA release in the context of cell death (discussed later in this Review), whereas West *et al* provided evidence that TFAM deficiency promotes mitochondrial stress and mis‐packaged mtDNA, resulting in their ejection into the cytoplasm where they bind and activate cGAS initiating a type I interferon response 39 (Fig 2). Of pathophysiological relevance, infection with Herpes simplex virus‐1 (HSV‐1) or vesicular stomatitis virus (VSV) results in mtDNA stress, TFAM depletion and mtDNA entrance into the cytoplasm. The cytoplasmic mtDNA is then sensed by cGAS, triggering cGAS‐STING signalling leading to the up‐regulation of a plethora of interferon genes, conferring an anti‐viral state on the cell. Importantly, *Tfam* ^+/−^ cells, which exhibit mtDNA stress, are more resistant to infection with HSV‐1 or VSV than wild‐type cells, as they have heightened ISG expression owing to mtDNA release. Mechanistically, the HSV‐1 virus encodes a nuclease, UL12.5, which localises to the mitochondria and degrades mtDNA, resulting in complete loss of mtDNA in infected cells 40, 41. Removal of mtDNA in infected cells does not appear to impact HSV replication 42. Furthermore, exonuclease activity is required for effective viral DNA production to maintain cell‐to‐cell infectivity, though whether this is related to UL12.5's mtDNA‐targeted nuclease activity is unknown 43.

Curiously, infection with RNA viruses, such as dengue virus, also elicits a cGAS‐STING response, despite cGAS being a DNA‐specific PRR 44. Several studies have now shown that dengue virus causes the release of predominantly oxidised mtDNA into the cytosol, where it can activate both cGAS 45, 46 and TLR9 47. Dengue virus has evolved strategies to circumvent cytosolic mtDNA‐induced cGAS signalling during infection by encoding proteases which target cGAS and STING for degradation, thus ensuring persistence of the virus 46, 48, 49.

Infection with the bacterial pathogen *Mycobacterium tuberculosis* triggers cGAS activation and subsequent IRF3‐dependent type I interferon response 50, 51, 52. This was assumed to be solely due to detection of mycobacterium DNA, but other studies have identified a role for mitochondrial stress and ensuing release of mtDNA into the cytoplasm 53. This observation is strain‐dependent but does propose a role for mitochondrial stress and dynamics on the *M. tuberculosis*‐induced release of mtDNA. Previous work has observed cytochrome *c* release from mitochondria in cells infected with *M. tuberculosis*, indicating that there may be a possible role for BAX/BAK‐dependent mitochondrial permeabilisation (discussed in detail later) in infection‐related mtDNA release 54 (Fig 2).

Pathogen‐infected cells often secrete IL‐1β due to inflammasome activation. A recent report by Aarreberg *et al* discovers a link between IL‐1β secretion in infected cells, which can then activate a cGAS‐STING‐dependent type I interferon response in surrounding bystander cells. Interestingly, IL‐1β stimulation of bystander cells increases mitochondrial mass, decreases mitochondrial membrane potential and induces mtDNA release 55. However, mtDNA release is observed in the absence of detectable cytochrome *c* release and cell death, suggesting that this is not the mechanism of mtDNA release, although it does not rule out limited mitochondrial permeabilisation seen by us and others in the context of infection (see below). This is not the first time IL‐1R signalling has been implicated in cell‐intrinsic defence 56, 57, 58, but it is the first to suggest that mtDNA release plays a key role in the initiation of cGAS‐STING signalling in the bystander cells.

### mtDNA activation of cGAS‐STING during cell death

**Figure 3:**
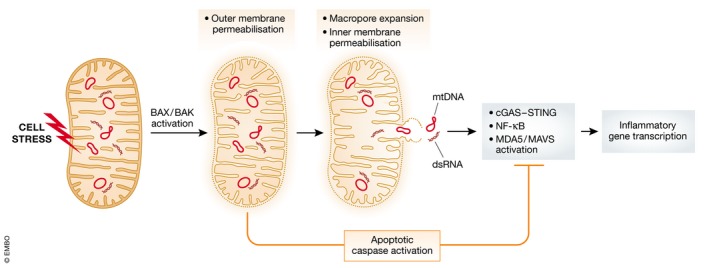
BAX/BAK‐dependent initiation of inflammation Following a pro‐apoptotic stress, BAX and BAK are activated leading to mitochondrial outer membrane permeabilisation. This enables the release of caspase‐activating proteins from the mitochondrial intermembrane space. Following this, macropores form on the mitochondrial outer membrane causing extrusion and permeabilisation of the inner membrane. This enables release of mtDNA. Mitochondrial double‐stranded RNA (dsRNA) can also be released. Collective release of these molecules triggers inflammation via MAVS, cGAS‐STING and NF‐κB. Caspase activity is anti‐inflammatory, in part, through direct cleavage and inactivation of inflammatory signalling molecules.

**Figure 4:**
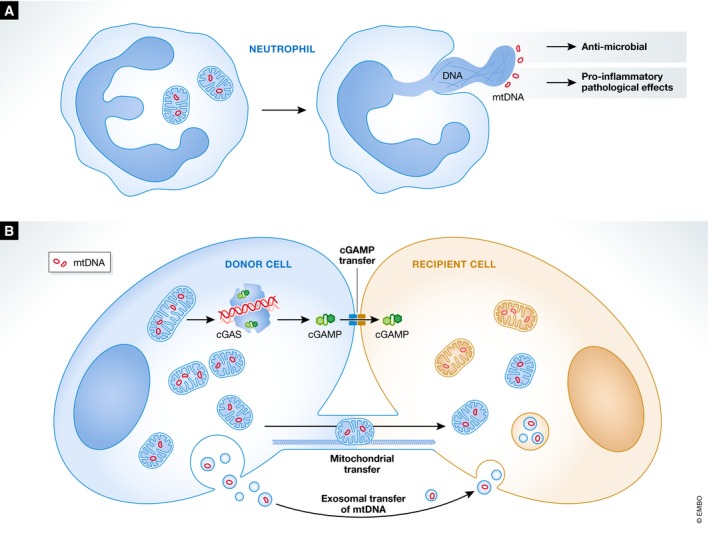
Non‐cell autonomous effects of mtDNA (A) Upon pathogen encounter, neutrophils can extrude DNA (both nuclear and mitochondrial) that forms an extracellular trap for extracellular microbes. Due to pro‐inflammatory properties, these DNA neutrophil extracellular traps (NETs) can also have pathological effects in diseases such as lupus. (B) mtDNA can transfer via exosomes or in intact mitochondria to neighbouring cells, impacting on the metabolism and survival of the recipient cell. Inflammatory responses to mtDNA can also have non‐cell autonomous effects. The cGAS‐induced secondary messenger cGAMP has been shown to transfer via gap junctions eliciting anti‐viral interferon responses in neighbouring cells.

During programmed cell death, the pro‐apoptotic proteins BAX and BAK permeabilise the mitochondrial outer membrane to allow the passage of pro‐apoptotic molecules to move from the inner membrane space into the cytosol, where they can initiate a caspase cascade, resulting in a rapid cell death 59. White *et al* and Rongvaux *et al* showed that in the absence of apoptotic caspase activation, mtDNA activates cGAS in a promiscuous manner, which *in vivo* leads to mildly elevated IFN‐β protein levels in blood, though a level sufficient to induce the expression of interferon‐stimulated genes 37, 38 (Fig 3). This suggests that apoptotic caspases play a crucial role in dampening type I interferon responses in dying cells, maintaining the “immune‐silent” nature of apoptosis (Fig 3). Further work has shown that apoptotic caspases directly cleave cGAS, IRF3 and mitochondrial anti‐viral signalling protein (MAVS), key proteins required for the production of type I interferon 60, supporting the notion that caspases dampen the immune response during cell death. High‐resolution imaging studies have further expanded our understanding of how mtDNA is released from the mitochondria during cell death. We and others recently showed that BAX and BAK can permeabilise the mitochondrial outer membrane, but in the context of caspase inhibition these pores grow dramatically, sufficient to allow inner membrane herniation and extrusion of mtDNA 61, 62, 63 (Fig 3). We found that under caspase‐inhibited conditions, mitochondrial permeabilisation leads to down‐regulation of inhibitor of apoptosis proteins (IAPs), NF‐κB‐inducing kinase (NIK) activation and an NF‐κB transcriptional program, in addition to mtDNA release‐induced cGAS‐STING activation 64. The cytokines and chemokines up‐regulated via NF‐κB after mitochondrial permeabilisation can serve to promote macrophage activation 64, 65. This leads to robust anti‐tumour effects, highlighting a potential therapeutic role for caspase inhibition in cancer treatment 64. Collectively, these results help to reconcile how predominantly cytosolic cGAS can be activated by mtDNA during cell death. Nevertheless, a number of unresolved questions remain. Firstly, is inner membrane permeabilisation a regulated process, and if so, how? A rapid inner membrane permeabilisation of sufficient size to allow the passage of small ions is observed minutes after outer membrane permeabilisation 61, but is insufficient to allow mtDNA nucleoid extrusion and is probably only transient, as inner membrane potential can be maintained after outer membrane permeabilisation 66, 67, 68, 69. Secondly, there are cell type differences in the degree of inner membrane permeabilisation, as different studies report varying degrees of mtDNA release during cell death 61, 62, implying that specific cell‐intrinsic factors play a role in inner membrane permeabilisation. Finally, the physiological relevance of cell death‐related mtDNA release is unknown. Most cell types undergo rapid and complete caspase‐dependent apoptosis *in vivo*, presumably limiting any potential for mtDNA‐driven inflammation during cell death. However, some cell types, for instance cardiomyocytes, display deficient caspase activity downstream of mitochondrial permeabilisation 70. Such cells might generate a greater type I anti‐viral interferon response after mitochondrial permeabilisation. Alternatively, cGAMP might transfer from apoptotic to healthy cells, serving as an “early warning” defence system, instructing healthy cells to transcribe genes important for their survival (Fig 4) 71, 72.

In addition to DNA, mitochondria also possess dsRNA which is known to be potently immunogenic 73. Mitochondrial dsRNA arises from transcription of both the heavy and lights strands of mtDNA; however, although the light strand is rapidly degraded the heavy strand is not, and nearly all the dsRNA detected in the cytoplasm are of mitochondrial origin. The mitochondrial helicase SUV3 and polynucleotide phosphorylase PNPase dampen the accumulation of dsRNA, but when these are depleted, dsRNA accumulates in the cytoplasm where it activates a type I interferon response driven by the dsRNA receptor MDA5 74. Silencing of BAX and BAK suppresses the type I interferon response, strongly suggesting that BAX/BAK‐dependent mitochondrial outer membrane permeabilisation is responsible for mitochondrial dsRNA escape into the cytoplasm 74 Furthermore, patients with mutations leading to a decrease in *PNPT1*, the gene that encodes PNPase protein, exhibit greater accumulation of dsRNA and elevated interferon levels in their serum 74.

Mitochondrial outer membrane permeabilisation is a rapid and complete event, spreading to all mitochondria in a cell. Following formation of BAX/BAK pores, pro‐apoptotic proteins such as cytochrome *c* are released from the intermembrane space where they initiate the caspase cascade, culminating in cell death. However, we have found that under conditions of sub‐lethal stress, a limited number of mitochondria in a cell can undergo permeabilisation, called minority MOMP, leading to genomic instability and transformation 75. A recent report by Brokatsky *et al* reveals a link between pathogen invasion and activation of mitochondrial cell death machinery 76. In this study, it was found that various pathogens can induce limited mitochondrial permeabilisation. It remains unclear how pathogens can trigger minority MOMP, but nevertheless they can, resulting in mtDNA release (presumably through BAX/BAK pores), stimulating cGAS‐STING activation and cytokine secretion 76.

How else might mtDNA be released from mitochondria? Another potential mechanism for mtDNA release from mitochondria is through the mitochondrial permeability transition pore (mPTP) 77, 78. The exact composition of the pore is unclear, although there seems to be consensus that cyclophilin D is present 79. The mPTP spans the mitochondrial inner membrane and forms in response to high mitochondrial calcium concentration and various other cellular stresses. However, the mPTP is predicted to only allow the efflux of molecules smaller than 1.5 kDa, much smaller than a mtDNA nucleoid 80, 81. In line with this, studies have shown that only fragments of mtDNA can pass through the mPTP 77, 82, 83. It remains possible that sustained opening of the pore can lead to swelling of the mitochondria and subsequent rupture of the inner membrane, which would permit the efflux of mtDNA into the cytoplasm. The involvement of mPTP in mtDNA release during cell death has been ruled out 61, but chitosan, a vaccine adjuvant, appears to induce a cGAS‐STING‐ and mPTP‐dependent type I interferon response. This is possibly due to mtDNA release, though a direct role for mtDNA has not been rigorously assessed 84. An intriguing recent report suggests that cells experiencing mitochondrial stress caused by the lack of mitochondrial endonuclease G release mtDNA through pores formed by oligomers of the voltage‐dependent anion channel (VDAC) 85. As mitochondrial DNA release is thought to play a role in the pathogenesis of lupus 86, 87, a role for VDAC pore formation was tested in an *in vivo* model of lupus‐like disease. Using the VDAC1 oligomerisation inhibitor VBIT‐4, the authors were able to reduce lupus‐like symptoms in lupus‐prone mice, providing a rationale to target VDAC‐mediated mtDNA release in this disease 85.

### Therapeutic targeting of mtDNA‐dependent cGAS‐STING activity

There is currently intense interest in the development of inhibitors and activators of the cGAS‐STING pathway, depending on the disease. In humans, the systemic inflammatory disease Aicardi–Goutières syndrome (AGS) is characterised by mutations in a number of different genes involved in DNA sensing 88. For example, TREX1, a DNA exonuclease, is frequently mutated in human patients with AGS and systemic lupus erythematosus (SLE) 89, 90, 91, and co‐deletion of cGAS, STING, Interferon‐α/β receptor (IFNAR) or IRF3 rescues this phenotype 92, 93, 94, 95, 96, 97, 98. Accumulation of cytosolic DNA appears to be a defining characteristic of AGS and SLE, as deletions in DNA‐ and RNA‐related genes including *SAMDH1*, a DNA exonuclease and *RnaseH2* are frequent 99, 100, 101, 102. Gain‐of‐function mutations in STING itself lead to an up‐regulation of type I interferon responses and lupus‐like symptoms in patients 103, 104. DNase II deficiency in humans leads to autoinflammation with increased type I IFN 105 and in mice causes arthritis 106. This is thought to be due to the lack of self‐DNA degradation in dead cells engulfed by macrophages resulting in sustained cGAS‐STING stimulation 98, 106, 107, and AIM2 inflammasome formation 108, 109 with a possible contribution of endosomal TLRs 108. Myocardial infarction (MI) is another condition known to involve a strong inflammatory component. King *et al* 110 showed that ischaemic cell death and engulfment by macrophages drives an IRF3‐dependent type I IFN response. Genetic or pharmacological disruption of cGAS‐STING signalling in mice improved their outcomes post‐MI, proposing this signalling axis as suitable for therapeutic intervention in patients 110, 111. While it is not clear if this is due to mtDNA release *per se*, increased mtDNA in plasma from patients with heart disease has been frequently observed 112, 113, 114. Clearly, inhibiting the cGAS‐STING pathway in these disease settings might be beneficial to patients. Small molecules targeting both cGAS 115, 116 and STING 117 have been developed, with STING antagonists emerging as the most promising. Blocking the IFNAR receptor to block interferon signalling in SLE patients had seemed like a viable therapeutic route; however, late‐stage clinical trials in this area have failed, prompting more investigation of how important interferon signalling is in the pathogenesis of SLE.

The ability to turn immunologically “cold” tumours “hot” and make them more responsive to immunotherapy is a desirable outcome in cancer treatment. Efficient T‐cell responses to tumour cells is a critical step to durable cancer treatment control 118. STING is required for spontaneous CD8^+^ T‐cell priming *in vivo* 119. Mechanistically, dying tumour cells transfer their DNA to antigen‐presenting dendritic cells when phagocytosed, eliciting cGAS‐STING‐IRF3 signalling leading to an anti‐tumour T‐cell response 119, 120, 121. Activation of STING by addition of exogenous cGAMP can also enhance anti‐tumour immunity after irradiation 120, the first evidence that therapeutic activation of STING may improve cancer therapy. This effect was later shown to be exclusive to dendritic cells over macrophages; blockade of the “don't eat‐me” signal CD47 results in increased tumour‐originated mtDNA in the cytosol of DCs and is required for the cross‐priming and type I IFN response mediated through cGAS 122. Dying tumour cells transfected with exogenous cytosolic DNA, viral DNA or cyclic dinucleotides (CDNs) have a greater capacity to activate STING signalling in antigen‐presenting cells, enhancing T‐cell priming and expansion of anti‐tumour T cells 123. Therefore, it is also possible that mtDNA may act as a STING activator in antigen‐presenting cells (APCs) under certain circumstances, for example when apoptotic caspases are inhibited. Another example of immune cell communication is in the interaction of T cells with antigen‐presenting dendritic cells. Upon formation of an immunological synapse between these two cell types, T cells shed extracellular vesicles (EVs) containing genomic and mtDNA. These EVs are taken up by the dendritic cell, triggering a cGAS‐STING‐dependent anti‐viral response, conferring resistance to subsequent viral infection 124. In the context of cancer treatment, it is plausible that apoptotic cell‐containing dendritic cells could stimulate a similar effect in T cells, generating longer lived dendritic cells for more durable treatment responses 125. Together, these data and many others provide a rationale for enhancing STING signalling in cancer treatment, and this is currently under active investigation 126, 127.

### mtDNA release, cGAS‐STING and neurodegeneration

Under normal, homeostatic conditions, damaged or stressed mitochondria are eliminated from the cell by a type of mitochondrial‐selective autophagy called mitophagy 128. Mutations in proteins involved in mitophagy pathways can contribute to neurodegeneration. This is perhaps best evidenced for PINK1/Parkin‐dependent mitophagy. For instance, loss‐of‐function mutations in the PINK1/Parkin pathway of mitophagy associate with early onset Parkinson's disease 129, 130, 131, 132, 133. In a simplified view, the kinase PINK1 is activated on dysfunctional mitochondria where it phosphorylates ubiquitin. Phospho‐ubiquitin allosterically activates the E3 ubiquitin ligase Parkin leading to enhanced mitochondrial ubiquitination that serves as an autophagic signal to remove the damaged mitochondrion 134, 135, 136. Parkinson's disease is associated with neuroinflammation 137, and the serum from Parkinson's patients is often enriched for pro‐inflammatory cytokines, including TNF, IL‐1β, IFNɣ and IL‐6 138, 139. However, many of the studies elucidating the mechanistic basis of Parkinson's have been performed in cultured cell lines, and despite much effort, the *in vivo* relevance of PINK1/Parkin‐mediated mitophagy was not well understood, particularly since mice that lack either PINK1 or Parkin exhibit no Parkinson's‐like disease phenotypes 140, 141, 142. Knowing that defective mitochondria can release innate immune‐activating DAMPs, Sliter *et al* 143 investigated the effect of exhaustive exercise or mtDNA mutation on inflammation. When challenged with exhaustive exercise, *Parkin* ^−/−^ or *Pink1* ^−/−^ mice displayed higher serum levels of pro‐inflammatory IL‐6 and IFN‐β when compared to wild‐type mice, in addition to increased levels of uncleared mitochondria. Remarkably, this could be completely rescued by deletion of STING or administering IFNAR‐blocking antibody to mice, strongly suggesting that mtDNA released from damaged mitochondria that are not cleared is responsible for the inflammation observed in Parkinson's patients. Interestingly, the authors also observed increased circulating mtDNA in *Parkin* ^−/−^ mice following exhaustive exercise, meaning that the mtDNA is not only extruded from mitochondria but also exits the cell. *Mutator* mice expressing a proofreading‐defective mtDNA polymerase (PolG) accumulate mutations in mtDNA, which instead of causing neurodegeneration results in dopaminergic neuron loss and defective movement. While no difference in inflammatory cytokine levels was noted between wild‐type, *Parkin* ^−/−^ or mutator mice, *Parkin* ^−/−^;*mutator* mice do have higher serum cytokine levels. Again, cytokine levels and the movement disorder could be completely rescued by co‐deletion of STING, reinforcing the cGAS‐STING axis as the major player in Parkinson's‐associated inflammation. However, further work is needed to elucidate the absolute requirement for mtDNA over nuclear DNA and the precise mechanism of how mtDNA is released from the mitochondria.

## mtDNA as an inflammasome activator

Inflammasomes are multi‐subunit complexes which form in response to exogenous PAMPs and DAMPs 144. One of four receptors—absent in melanoma 2 (AIM2), NOD, LRR and Pyrin domain‐containing protein 1 (NLRP1), NLRP3 or NLR family CARD domain‐containing protein 4 (NLRC4), bind to the adaptor molecule ASC forming a platform for the dimerisation, autoprocessing and activation of caspase‐1. Active caspase‐1 can then process pro‐IL‐1β and pro‐IL‐18 into their mature form so they can be secreted (see Fig 1). The first report of mtDNA acting as an activator of inflammasomes came in 2011 when Nakahira *et al* 145 reported that depletion of proteins involved in autophagy leads to an accumulation of dysfunctional, persistent mitochondria exhibiting excessive ROS. These mitochondria were more prone to extrude mtDNA into the cytoplasm upon stimulation with lipopolysaccharide (LPS) or ATP, dependent on the ability to from NLRP3 inflammasomes. Interestingly, Nakahira *et al* suggested that as well as acting downstream of mtDNA release, NLRP3 also acts upstream, to facilitate mPTP formation on the mitochondria allowing mtDNA release. However, as already discussed, whether mPTP is sufficient to allow mtDNA translocation from the mitochondrial matrix into the cytoplasm is debatable. Extending this work, the following year Shimada *et al* 146 reported that during macrophage apoptosis mtDNA is released and binds NLRP3. Notably, NLRP3 appears to have a preference for oxidised mtDNA, clarifying the observations that ROS plays a crucial role in inflammasome activation 147. Linking these observations, deletion of the autophagy receptor p62 prevents mitophagic clearance of mitochondria damaged by NLRP3 agonists, exacerbating inflammasome formation and IL‐1β secretion 148. More recent work has pointed to newly synthesised, oxidised mtDNA as the species which binds NLRP3 149. Zhong *et al* discovered that levels of the mitochondrial deoxyribonucleotide kinase CMPK2 increase upon LPS stimulation. CMPK2 catalyses a step in the synthesis of the nucleotide cytidine triphosphate, which is rate‐limiting for mtDNA synthesis. Elevated dCTP levels in turn increase mtDNA replication, which is oxidised by ROS and released into the cytoplasm where it can activate NLRP3 and stimulate IL‐1β secretion. However, the role of NLRP3 as a direct sensor of DNA is contentious, as many disparate signals have been reported as the common signal for NLRP3 activation 144. Indeed, recent work from the Chen laboratory has shown that dispersal of the *trans*‐Golgi network following K+ efflux is the likely common trigger 150.

Supporting the notion that inflammasomes and caspase activity can act upstream of mtDNA release, there are reports that caspases cause mitochondrial damage. For example, inflammasome‐activated caspase‐1 has been reported to damage mitochondria and promote the release of cytochrome *c*, indicative of mitochondrial outer membrane permeabilisation 151. The authors suggest that this is due to mPTP formation, although a role for BAX and BAK was not rigorously assessed in this work. Impairment of mitophagy was also implicated, as Parkin was found to be a substrate of caspase‐1 in macrophages, leading to an accumulation of damaged, ROS‐producing macrophages 151. Furthermore, during infection‐related ER stress, NLRP3 (but not the adaptor protein ASC or caspase‐1) is involved in caspase‐2 activation and cleavage of the pro‐apoptotic protein BID, promoting mitochondrial permeabilisation 152.

## Neutrophil extracellular traps

So far, we have mainly discussed the cell autonomous role of mtDNA release; however, it is becoming clear that mtDNA can also be extruded from the mitochondria, into the cytoplasm and outward further into the extracellular space. One interesting example of this is in the generation of neutrophil extracellular traps, and in particular the role of mtDNA in their formation (Fig 4).

Neutrophils are the first line of attack in infection, capable of engulfing pathogens and degranulating, the process of releasing soluble anti‐microbials. In 2004, Brinkmann and colleagues discovered that upon stimulation with IL‐8, phorbol myristate acetate (PMA) or LPS, neutrophils extruded vast fibrous networks, which they termed neutrophil extracellular traps (NETs) 153. Analysis of these NETs showed that they contained a variety of microbial‐killing proteins, including elastase, cathepsin G and myeloperoxidase. However, they also contain DNA, as noted by reactivity with antibodies against histones and DNA intercalating dyes. Successive work showed that NETs were also enriched for mtDNA 154, 155, 156. NET formation has been well studied in patients with systemic lupus erythematosus (SLE), an autoimmune condition hallmarked by the appearance of autoantibodies against dsDNA and RNA‐protein complexes, resulting in elevated type I interferon responses. A number of studies show that mtDNA is part of NETs formed in SLE. Caielli *et al* 87 found that in healthy neutrophils, mitochondria with oxidative damage are removed not via mitophagy, but by extruding their mitochondrial matrix contents, including TFAM‐mtDNA nucleoids, into the extracellular space. These TFAM‐mtDNA nucleoids are devoid of oxidised DNA, and so do not activate plasmacytoid dendritic cells (pDCs) and thus are not immunogenic. Healthy neutrophils remove oxidised mtDNA by signalling PKA phosphorylation of TFAM which initiates its degradation and by shuttling oxidised mtDNA into lysosomes. In contrast, neutrophils in SLE have reduced PKA activation and so do not degrade TFAM as efficiently, leading to the extrusion of immunogenic oxidised mtDNA 87. Another report reveals ROS to be an important mediator for neutrophils to produce oxidised mtDNA‐containing NETs in response to stimulation by ribonucleotide immune complexes (RNP ICs) 86. The authors also found that injecting this DNA was pro‐inflammatory and dependent on the STING pathway revealing a dual role for mitochondria in providing the source of DNA for NETs and oxidising it for maximal interferogenic response in SLE 86 (Fig 4). Sustained IFNα signalling in SLE is also known to deregulate mitochondrial metabolism in monocytes, leading to reduced autophagy and an accumulation of mtDNA in the cytoplasm. This leads to cGAS‐STING activation which promotes secretion of TNF and IL‐6 and the expansion of self‐DNA autoreactive lymphocytes 157. It is now also appreciated that other cell types, including lymphocytes and eosinophils, can secrete mtDNA‐containing webs which act to prime type I interferon responses in peripheral blood mononuclear cells 158, 159.

Nuclear DNA is prepared for expulsion as NETs through a highly regulated process involving decondensation of chromatin and citrullination of histones. Furthermore, plasma membrane permeabilisation is also regulated, inevitably leading to cell death. Within minutes of stimulation, neutrophils rapidly produce NETs, whereas the death of neutrophils (dubbed “NETosis”) occurs ~2 h after 160. While these two phenomena are often conflated in the literature, the timing argues against a general lytic mechanism of mtDNA release. In fact, release of mtDNA as NETs seems to be energy‐dependent 161. The precise mechanism of mtDNA escape during NET formation remains to be elucidated; one possibility is that it may be due to BAX/BAK pore formation on the mitochondrial outer membrane 61, 62, although this seems unlikely as this would induce a rapid cell death.

## mtDNA and TLR9

The Toll‐like family of receptors (TLR) recognise a plethora of different bacterial features to instigate innate immunity. TLR9 recognises hypomethylated CpG motifs found in bacteria. TLR9 is expressed primarily in monocytes, macrophages, plasmocytoid dendritic cells and B lymphocytes. In resting cells, TLR9 resides on the endoplasmic reticulum, but recognition of DNA occurs in the endolysosomes (see Fig 1) 162, 163, 164, 165. DNA‐bound TLR9 recruits MyD88 which activates MAPK and NF‐κB, inducing an inflammatory response. In common with bacterial DNA, mtDNA is hypomethylated at CpG motifs, making it a potent activator of TLR9 166, 167. mtDNA detection by TLR9 was first noted in 2010 by Zhang *et al*, who observed that during systemic inflammatory response syndrome (SIRS) mtDNA was released into the blood where it can activate TLR9 on neutrophils 168, 169. In the heart, autophagy is required to remove damaged mitochondria and maintain heart function during hemodynamic stress 170. However, when DNase II, a lysosomal DNase, is deleted from cardiac cells, the mice succumb faster following heart pressure overload 171. Delving deeper into the mechanism, the authors found that this was due to an increase in mtDNA which has escaped degradation, thus activating a TLR9‐dependent inflammatory response 171. Mitochondrial DNA released from dying cells or as part of NETs can form a complex with the anti‐microbial peptide LL‐37. This mtDNA:LL‐37 complex evades degradation by DNase II and can activate TLR9 on pDCs, neutrophils and endothelial cells to exacerbate atherosclerosis 172. High‐mobility group box 1 (HMGB1) is a DNA‐binding protein released from necrotic 173 and cytokine‐stimulated cells 174. HMBG1 binds a receptor, called RAGE, leading to inflammatory signalling. In particular, HMGB1 has been shown to be released from pDCs following stimulation with CpG oligodeoxynucleotides (ODNs). CpG‐ODNs can bind and activate TLR9, but when complexed with HMGB1 the inflammatory response is augmented through HMGB1 activation of RAGE 175. In an analogous manner, TFAM co‐operates with mtDNA released from necrotic cells to increase pro‐inflammatory signalling in pDCs through RAGE and TLR9 176, 177.

TLR9 has been particularly well studied in liver pathologies. In liver cancer, hypoxia triggers the translocation of mtDNA and HMGB1 into the cytoplasm of cancer cells to activate TLR9, resulting in tumour cell proliferation 178. TLR9 is crucial for the development of acetaminophen‐induced hepatotoxicity 179 and fibrosis 180. Development of non‐alcoholic steatohepatitis (NASH) involves innate immunity, with hepatic stellate cells and macrophage‐like Kupffer cells being particularly relevant. Mice fed a choline‐deficient amino acid‐defined diet develop NASH, whereas TLR9^−/−^ mice do not, implicating TLR9 as a requirement for NASH development 181. The precise ligand for TLR9‐derived liver disease was poorly understood, although the observation that NASH patients had higher mitochondrial mass, but reduced respiration, suggested that mitochondria may play a role 182. In line with these observations, Garcia‐Martinez *et al* 183 found that mice and human patients with NASH exhibited higher levels of oxidised mtDNA in hepatocytes and plasma. As the oxidisation of mtDNA increases its ability to activate TLR9, the authors confirmed that was the case. Importantly, mice dosed with a TLR9 antagonist displayed reduced symptoms of NASH, validating the importance of mtDNA release and TLR9 signalling in the pathogenesis of NASH 183. NASH is characterised by different forms of cell death, most prominently apoptosis 184 and necrosis 185, 186. In hepatocytes, mitochondrial permeabilisation results in an increase of DNase II activity, and knockdown of DNase II switches the mode of cell death to a RIP1‐dependent non‐apoptotic form 187. Importantly, this is due to the release of mtDNA after mitochondrial permeabilisation, which triggers TLR9 signalling and subsequent IFNβ secretion. In mice fed a high‐fat diet, a model of NASH, DNase II activity is diminished, providing a mechanistic link as to how necrosis of hepatocytes can augment NASH symptoms in patients 187. It is unclear why the release of mtDNA triggers either cGAS‐STING or TLR9 signalling in different studies; however, it may be due to different cell types, length or oxidation status of mtDNA, activity of DNA nucleases or different cellular compartments.

Mutations in OPA1, a protein required for mitochondrial inner membrane fusion and cristae formation, have been reported to cause mtDNA instability 188, 189, 190, 191. Deletion of OPA1 in skeletal muscle, a tissue with high metabolic demands, predictably results in mitochondrial dysfunction, mtDNA stress and inflammation leading to reduced growth and early death in mice 192, 193. Interestingly however, OPA1 deletion leads to disruption of mitophagy due to impaired autophagic flux resulting in higher levels of dysfunctional mitochondria in these tissues 194. When mtDNA localisation was examined, following OPA1 deletion there is high co‐localisation of mtDNA and TLR9, implicating TLR9 as the driver of OPA1‐deletion inflammation 194.

## Transfer of mtDNA between cells

So far, this Review has mainly focussed on the cell‐intrinsic biological effects of mtDNA release. However, it is possible that released mtDNA nucleoids could move from one cell to another, thus “spreading” the inflammatory signal across a population of cells. It is now well established that mitochondria, including mtDNA, can be transferred between cells (Fig 4). A seminal study in 1989 was the first to describe such a phenomena, where cells devoid of mtDNA (ρ^0^ cells) and thus lacking respiratory competence could be repopulated with mitochondria from other cell lines 195. More recent work has shown that following stroke, whole mitochondria can be transferred from astrocytes to neurons, a process proven to be beneficial to recovery 196. In cancer models, ρ^0^ cells have delayed tumour growth, likely due to defects in energy production. Horizontal transfer of mitochondria from cells in the tumour microenvironment restored respiration in ρ^0^ cells and instigated tumour growth 197. Horizontal transfer of mitochondria could occur through a number of different mechanisms. Firstly, cancer cells can form tunnelling nanotubes with cells in the tumour microenvironment, through which cytoplasmic contents, including mitochondria, can be transferred 198. Tunnelling nanotubes form between endothelial cells and cancer cells to transfer mitochondria, conferring chemoresistance to the cancer cell 199 but also between early apoptotic cells and healthy cells, where mitochondrial transfer can reverse apoptosis 200. Secondly, mtDNA has been proposed to be packaged into extracellular vesicles (EVs). Specifically, cancer‐associated fibroblasts can package entire mitochondrial genomes into EVs which then fuse with cancer cells to transfer mtDNA. Importantly, the size of these EVs, ~100 nm, is far below the size of a mitochondria, so making it unlikely that an entire mitochondria is transferred in this manner 201. However, mtDNA nucleoids are within these size constraints 21. It is important to note that other studies see transfer of entire mitochondria between cells, and so whether these or just mtDNA genomes are transferred is controversial 202. Thirdly, mitochondria can be directly transferred between cells through connexin 43 gap junctions, as had been seen between bone marrow‐derived stromal cells and pulmonary alveoli during lung injury 203. Interestingly, transplanting tumour or embryonic stem cells into hosts with the same nuclear DNA background but different mtDNA from allogenic mouse strains resulted in rejection 204. Mechanistically, this is dependent on MyD88, the adaptor molecule required for TLR9 signalling, suggesting that TLR9 may be the PRR in this situation 204. However, whether or how mtDNA is released from these cells is unknown, but it is clear that allogenic mtDNA can trigger innate immune pathways. This hints at the intriguing notion that inflammation could spread between cells via detection of mtDNA, perhaps through connexin 43 gap junctions, in a similar manner to the observation that cGAMP can transfer to activate STING in neighbouring cells 71 (Fig 4). Contrary to this is data showing that cell‐free mtDNA (for example, as seen in sepsis) can actually suppress inflammation 205. Increased serum concentration of mtDNA is associated with a poorer outcome in sepsis patients, and injection of mtDNA in mice suppresses the adaptive immune response in a TLR9‐dependent manner 205. Immunosuppressive markers, such as an increase in PD‐L1 expression in the spleen, are seen in mice injected with mtDNA, which is reflective of what is seen in sepsis patients 205. Clearly, there is conflicting data on the immunostimulatory or immunosuppressive role of cell‐free mtDNA, which may depend on pathophysiological context; nevertheless, release of mtDNA appears to potently affect the immune system.

## Conclusions and future perspectives

Mitochondria are multi‐faceted organelles orchestrating key events in both life and death. They represent a rich source of DAMPs which can potently trigger the innate immune system, such as ATP, formyl peptides and mtDNA. Possibly stemming from its bacterial origin, mtDNA is particularly effective at initiating inflammatory and anti‐viral signalling.

The last number of years has seen an explosion in interest in how mitochondria initiate innate immunity in the context of pathogen invasion, cell death and pathology. However, many of these studies leave us with unresolved questions as to precisely how mtDNA is extruded from the mitochondria. In the context of cell death, it is now clear that BAX and BAK form the pores on the mitochondrial outer membrane through which the inner membrane herniates, leading to mtDNA release, although how the inner membrane permeabilises is as yet not fully resolved 61, 62. Many other studies have suggested that the mPTP is involved in various contexts, but again this is controversial. Clearly, further investigation is required, whether to determine a more universal role for BAX/BAK‐dependent mtDNA release, utilising our current knowledge of the nature of the mPTP, or whether an altogether unknown mechanism is involved.

It is also apparent that cellular context will determine how mtDNA causes inflammation. cGAS‐STING signalling seems to be widely available across most cell types, a notable exception being some transformed cells. However, TLR9 protein expression appears to be restricted to immune cells, as does expression of inflammasome components. Perhaps most interesting will be determining what the outcomes of triggering innate immunity with cytosolic or cell‐free mtDNA are. For example, in the context of cell death, does production of cGAMP in apoptotic cells transfer to healthy apoptotic cells via gap junctions to promote a death‐resistant state, in a manner similar to what has been observed in astrocytes 71? Pathogen invasion stimulates a limited degree of mtDNA release by hijacking the apoptotic machinery, so it is plausible to see how this might act as a cell‐intrinsic warning system, but it will be fascinating to understand how this functions in the context of a whole tissue. Furthermore, can we leverage what we have learnt about anti‐viral signalling during cell death to enhance anti‐cancer therapy by inhibiting caspases? Likewise, will our understanding of how mtDNA and STING function in neurodegeneration lead to novel therapeutic strategies to enhance healthy ageing 143? Along these lines, mitochondrial dysfunction has been shown to induce a specific form of senescence termed MiDAS (mitochondrial dysfunction‐associated senescence) 206—given the links between ageing, senescence and inflammation, it is tempting to hypothesise that mtDNA plays a role in the initiation of this phenotype.

A broad spectrum of pathologies, from cancer, to autoimmunity and ageing all have aberrant mtDNA release as a driver or contributor of disease. Future work aimed at understanding how mtDNA is involved will no doubt afford us new therapeutic avenues with which to treat patients.

**In need of answers**

- Why do different tissue and cell types respond to cytosolic mtDNA through different pathways?
- Can mtDNA release be harnessed therapeutically for treatment of inflammatory diseases or cancer?
- Where is cGAS located in the cell?
- What are the physiological, non‐lethal effects of mtDNA release into the cytoplasm?

### Conflict of interest

The authors declare that they have no conflict of interest.
